# Follicle Stimulating Hormone and Anti-Müllerian Hormone per Oocyte in Predicting in vitro Fertilization Pregnancy in High Responders: A Cohort Study

**DOI:** 10.1371/journal.pone.0034290

**Published:** 2012-04-24

**Authors:** Andrea Weghofer, Ann Kim, David H. Barad, Norbert Gleicher

**Affiliations:** 1 Department of Obstetrics and Gynecology, Medical University Vienna, Vienna, Austria; 2 The Center for Human Reproduction and The Foundation for Reproductive Medicine, New York, New York, United States of America; 3 Departments of Epidemiology and Social Medicine and Obstetrics, Gynecology and Women’s Health, Albert Einstein College of Medicine, Bronx, New York, United States of America; 4 Department of Obstetrics, Gynecology and Reproductive Sciences, Yale University School of Medicine, New Haven, Connecticut, United States of America; Baylor College of Medicine, United States of America

## Abstract

**Background:**

Follicle stimulating hormone (FSH) and Anti-Müllerian hormone (AMH) are utilized to differentiate between good and poor response to controlled ovarian hyperstimulation. Their respective roles in defining functional ovarian reserve remain, however, to be elucidated. To better understand those we investigated AMH and FSH per oocyte retrieved (AMHo and FSHo).

**Methodology/Principal Findings:**

Three-hundred and ninety-six women, undergoing first in vitro fertilization cycles, were retrospectively evaluated. Women with oocyte yields >75^th^ percentile for their age group were identified as high responders. In a series of logistic regression analyses, AMHo and FSHo levels were then evaluated as predictive factors for pregnancy potential in high responders. Patients presented with a mean age of 38.0±5.0 years, mean baseline FSH of 11.8±8.7 mIU/mL and mean AMH of 1.6±2.1 ng/mL. Those 88 women, who qualified as high responders, showed mean FSH of 9.7±6.5 mIU/mL, AMH of 3.1±3.1 ng/mL and oocyte yields of 15.8±7.1. Baseline FSH and AMH did not predict pregnancy in high responders. However, a statistically significant association between FSHo and pregnancy was observed in high responders, both after univariate regression (p = 0.02) and when adjusted for age, percentage of usable embryos, and number of embryos transferred (p = 0.03). Rate of useable embryos also significantly affected pregnancy outcome independently of FSHo (p = 0.01). AMHo was also associated with clinical pregnancy chances in high responders (p = 0.03) and remained significant when adjusted for usable embryos and number of embryos transferred (p = 0.04).

**Conclusions:**

AMHo and FSHo are predictive of pregnancy potential in high responders, but likely reflect different responsibilities in recruitment and maturation of growing follicle cohorts.

## Introduction

Reproductive senescence is closely associated with the depletion of the ovarian follicle pool. The decline of functional ovarian reserve (FOR), i.e. the cohort of small antral follicles, appears to reflect this aging process [1], [2]. Functional ovarian reserve is strongly correlated with a variety of measurements, including follicle stimulating hormone (FSH) [3], [4], anti-Müllerian hormone (AMH) [5], [6] and antral counts [7]. Different assessment tools, though, subtly, represent different components of FOR [8].

AMH has recently attracted most attention in predicting ovarian response to controlled hyperstimulation [9], [10]. FSH, in contrast, has proven less successful in predicting large oocyte yields and hyperstimulation risk [10]. AMH levels are, therefore, increasingly used to individually tailor and adjust gonadotrophin dosage in assisted reproduction [11], [12], [13]. Though AMH and other ovarian function markers have proven helpful to predict oocyte yield, their significance in the prediction of egg quality and, consecutively, pregnancy, remains controversial [14], [15], [16].

AMH is exclusively produced by granulosa cells of small preantral and early antral follicles [17]. A statistical association with number of oocytes, therefore, does not surprise. AMH, however, is no longer produced at more mature follicle stages. Continuous association with egg/embryo quality, therefore, would be more surprising. FSH, in contrast, based on animal data, appears to have influence on follicle maturation from recruitment until full maturity [18].

In an attempt to better elucidate the association between FSH and AMH and oocyte/embryo quantity/quality, we decided to explore mathematical models of FSH and AMH levels per retrieved oocyte (FSHo and AMHo) as potentially new tools in improving assessments of functional ovarian reserve. In the assumption that any potential qualitative aspect of AMH’s (and potentially also FSH’s) predictive capacity may only become apparent in the presence of large enough oocyte yields, we restricted the study cohort to high responders.

## Methods

### Ethics Statement

Here presented data only involved retrospective review of medical records. Patients at our center sign at initial consultation an informed consent, which allows for such reviews if the patient’s medical record remains confidential and her identity protected. These conditions were met in this case, allowing for expedited approval by the Institutional Review Board (IRB).

### Participants

Between January 2005 and July 2011, 396 consecutive patients, who underwent at least one in vitro fertilization cycle at the Center for Human Reproduction in New York, N.Y, were retrospectively evaluated. To preclude the introduction of a potential bias on pregnancy chances, only first fresh treatment cycles, performed at our center, were considered for analysis. To identify high, normal and low responders, the dataset was stratified according to female age into the following groups: ≤34, 35–37, 38–40, 41–42, and ≥43 years, and by oocyte yields. Patients with oocyte yields above the 75^th^ percentile of their age group were coded as high responders. Normal responders had oocyte yields between the 25^th^ and the 75^th^ percentile of their age group and patients with oocyte yields below the 25^th^ percentile of their age group were coded as low responders. We previously investigated FSHo and AMHo in a similar patient group of 392 infertile women, with over 60 percent afflicted by diminished ovarian reserve [19]. This study, therefore, focused on high responders.

### Description of Procedures Undertaken

Baseline serum FSH and AMH were measured at cycle day 2 or 3 prior to cycle start. Serum AMH was obtained using the Diagnostics System Laboratories (DSL) assay by Beckman Coulter Inc., Brea, CA. The assay for serum AMH involved an enzymatically amplified two-site immunoassay, DSL-10-14400 active MIS/AMH ELISA. According to the manufacturer’s manual, the sensitivity of the assay was 0.025 µg/L with intra-assay variation <15% [20]. Serum FSH testing was performed in our clinical endocrine laboratory. All assays were performed on an AIA-600II (TOSOH Bioscience, Inc, Tokyo, Japan). The coefficient of variation of the assays, as determined by summation of semiannual quality control proficiency testing over this entire period, has been 8.1%.

All women underwent gonadotropin releasing hormone agonist (GnRh agonist) cyclesincluding stimulation with follicle stimulating hormone (FSH) and human menopausal gonadotropin (hMG) [21]. Women with age-specific normal ovarian function (i.e., normal age-specific AMH and FSH levels) received long protocol stimulation, while women with diminished ovarian reserve received microdose agonist stimulation [3], [22]. Ovulation induction and oocyte retrieval were performed when at least two follicles reached a diameter of 18 mm. The diagnosis of a clinical pregnancy was established by the presence of a fetal heartbeat on vaginal ultrasound.

In order to assess oocyte-specific AMH levels, the number of oocytes retrieved was divided by AMH (AMHo). In our prior manuscript, AMHo was calculated inversely (i.e., AMH divided by oocyte number). This calculation, however, produced very small numbers. We, therefore, decided to adapt this calculation. Oocyte-specific FSH was calculated as FSH per oocyte (FSHo). In order to determine the percentage of usable embryos, the sum of embryos transferred and cryopreserved was divided by total oocytes retrieved. Results were multiplied by 100 to establish percentages.

### Statistical Methods

A series of logistic regression was then performed to determine whether AMHo, FSHo and the percentage of useable embryos per oocyte yield predict clinical pregnancy in high responder patients. Statistical analysis was undertaken using SPSS 18.0 (SPSS, Chicago, IL). Baseline characteristics of patients were compared using t-tests. Outcome parameters are presented as proportions. The equation to predict the probability of clinical pregnancy per patient based on FSHo and AMHo, derived via logistic regression, is reported as Logit P. A P-value<0.05 was considered statistically significant. Continuous values are presented as mean ±SD.

## Results

Table 1 summarizes patient and cycle characteristics. The mean age for all 396 women was 38.0±5.0 years; mean baseline FSH was 11.8±8.7 mIU/mL and AMH was 1.6±2.1 ng/mL. Infertility diagnoses were: male factor infertility in 29.5%, tubal infertility in 23.9%, PCOS in 18.2% and diminished ovarian reserve in 45.5% (some patients carried more than one primary diagnosis).

**Table 1:** Patient characteristics and cycle parameters in 396 women according to their response to controlled ovarian hyperstimulation.

	high responders	normal responders	low responders
	(n = 88)	(n = 235)	(n = 73)
**Female age (years)**	37.9_a_±4.6	37.2_a_±5.2	41.0_b_±3.5
**AMH (ng/mL)**	3.1_a_±3.1	1.4_b_±1.5	0.4_c_±0.5
**Baseline FSH (mIU/mL)**	9.7_a_±6.5	11.1_a_±7.7	16.5_b_±12.1
**BMI (kg/m^2^)**	25.6_a_±5.8	24.5_ab_±5.0	23.5_b_±4.3
**FSH dosage (IU/ml)**	4539_a_±2534	5689_b_±2462	7291_c_±2081
**No. oocytes retrieved**	15.8_a_±7.1	7.2_b_±3.6	1.5_c_±0.5
**No. embryos transferred**	2.9_a_±0.8	2.5_b_±0.9	1.3_c_±0.4
**No. embryos cryopreserved**	4.7_a_±4.9	1.3_b_±2.3	0.0_c_±0.0
**Percentage of usable embryos per oocyte yield (%)**	50.4%_a_	52.4%_a_	86.3%_b_
**AMHo** *	11.1_a_±21.8	11.7_a_±16.0	10.7_a_±23.3
FSHo**	0.9_a_±1.3	2.1_b_±2.1	12.2_c_±11.0
**Clinical pregnancy (%)**	34.1%	28.5%	8.2%

*Values are presented as means ±SD.*

*Means in a row that do not shoare subscripts differ at p<0.05 in the Bonferroni comparison.*

* oocytes per AMH; ** FSH per oocyte.

Out of 396 women, 88 patients qualified as high responders. They showed mean FSH of 9.7±6.5 mIU/mL and mean AMH of 3.1±3.1 ng/mL; 8.1±6.4 average oocytes were retrieved in the whole cohort, with high responders producing 15.8±7.1 oocytes. Indications for fertility treatment had no significant impact on pregnancy in high responders. However, when differences in FSHo and AMHo according to indication for fertility treatment were evaluated, there were statistically significant differences in the median FSHo and AMHo values among high responders with and without PCOS (p = 0.017 and p = 0.019, respectively) and with and without diminished ovarian reserve (p = 0.001 and p = 0.004, respectively). There was also a statistically significant difference observed in FSHo between patients with and without tubal factor (p = 0.003).

**Figure 1:**
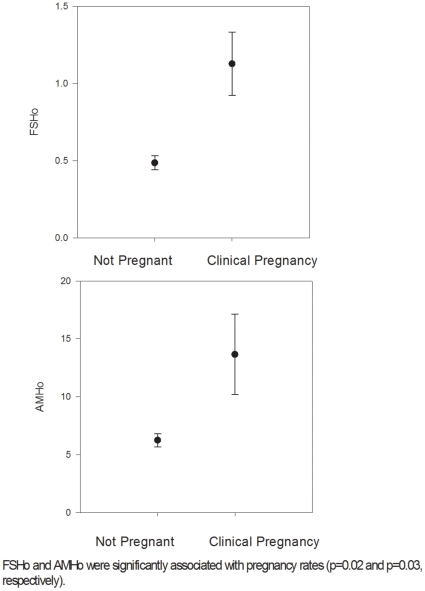
FSHo and AMHo and pregnancy potential in high responder patients.

A total of 103 clinical pregnancies were established, a clinical pregnancy rate of 8.2% in poor responders, of 28.5% in normal responders and of 34.1% in women with high age-specific oocyte yields. The relationship between ovarian response and clinical pregnancy was statistically significant (χ^2^ (2, n = 396) = 15.76, p<0.001). Univariate regression analysis revealed a statistically significant association between FSHo and pregnancy chance in high responders (ß−1.82; SE±0.75; p = 0.02; Logit P = 0.509−(1.816 * FSHo)) (Figure 1a). Total FSH, however, failed to demonstrate such an association.

When the effect of FSHo was adjusted for estradiol levels at ovulation induction, FSHo’s effect on pregnancy remained significant (β = −0.116, SE±0.057, p = 0.042). We then evaluated the effect of FSHo on pregnancy, adjusting for diminished ovarian reserve, PCOS and tubal factor in separate logistic regression models (i.e., one model for each diagnosis). FSHo’s effect on pregnancy also remained significant after adjusting for infertility diagnoses (p = 0.014, p = 0.026, and p = 0.013, respectively). When the effect of FSHo on pregnancy potential was adjusted for age, percentage of usable embryos and number of embryos transferred, FSHo’s association with pregnancy also remained significant (ß−1.81; SE±0.84; p = 0.03). In this model, the rate of useable embryos per oocyte yield also had, independent of FSHo, a significant effect on clinical pregnancy outcome (ß 0.03; SE±0.01; p = 0.01).

AMHo behaved similarily to FSHo. In univariate regression, AMHo was also significantly associated with clinical pregnancy rates in high responders (ß−0.12; SE±0.06; p = .03; Logit P = 0.310−(0.123 * AMHo)), while total AMH failed to show such association (Figure 1b). We then performed a series of logistic regressions to control for potential confounders: The effect of AMHo remained significant when adjusted for estradiol levels at ovulation induction (β = −1.768, SE±0.783, p = 0.024). We then evaluated the effect of AMHo on pregnancy, adjusting for diminished ovarian reserve and PCOS in two separate logistic regression models (i.e., one model for each diagnosis). AMHo’s effect on pregnancy also remained significant after adjusting for infertility diagnoses (p = 0.026 and p = 0.043, respectively). The significant effect of AMHo on pregnancy potential in high responders also remained significant when adjusted for usable embryos and number of embryos transferred (ß−0.12; SE±0.06; p = 0.04). When adjusting for age, usable embryos per oocyte yield and embryos transferred, usable embryos significantly predicted pregnancy chance in high responders (ß 0.03; SE±0.01; p = 0.01), while AMHo failed to reach significance (p = 0.10).

## Discussion

We recently reported the first use of FSHo and AMHo in infertile women, with a majority afflicted by diminished ovarian reserve [19]. In that cohort of women with limited amounts of embryos for transfer, FSHo, but not AMHo, was statistically associated with pregnancy chances. Here, in high responders, both, FSHo and AMHo, are predictive of pregnancy chances, while total FSH and AMH failed to demonstrate such associations. To correctly interpret these findings, AMH’s and FSH’s varying potentials in predicting oocyte *quantity* and *quality* should be considered: Because of its inhibitory effect on follicle recruitment, AMH levels rise in clinical situations associated with active recruitment, like polycystic ovary syndrome [23], and are low when recruitment is inactive, like in pregnancy [24], with use of oral contraceptives [25] or in women with diminished ovarian reserve [26], [27]. If one keeps in mind that AMH is produced within each follicle, and only during times of early follicle maturation, it should not surprise that AMH levels rather correspond to egg numbers than to egg quality [28], as demonstrated by our data showing that AMH did correlate with oocyte yield and not with pregnancy.

AMHo, in contrast, showed a significant association with clinical pregnancy rates in high responders (p = 0.03), in women with sufficient embryos for transfer. This association remained significant when adjusted for various impacts of excessive oocyte yield: peak estradiol levels (p = 0.04), number of embryos transferred and usable embryos (p = 0.04). AMHo, thus, appears to demonstrate predictive ability for pregnancy and, therefore, oocyte quality, once oocyte numbers no longer matter, while AMHo failed to do so in a previous cohort of women with diminished ovarian function [19]. Under such ideal circumstances, differences in pregnancy potential might reflect granulosa cell function. These assumptions are supported by our data that demonstrated a significant impact of different indications for fertility treatments (i.e., PCOS and DOR) on AMHo levels and may shed further light on the impact of oocyte quality on pregnancy potential in women with PCOS: Among our group of high responders, women with higher amounts of AMH per oocyte (i.e. lower AMHo) were more likely to achieve pregnancy. This, however, implicates that among high responders with comparable AMH levels, women with excessive oocyte yields experience lower pregnancy chances. Since we controlled for the number of embryos transferred and analysed only first cycles, our findings suggest that, among high responders, patients with excessive oocyte yields are more likely to have embryos with impaired implantation potential than their high responder counterparts. AMHo amongst high responders, thus, potentially defines two new PCO sub-phenotypes, one with high oocyte yields and better pregnancy chances and the other with excessive oocyte yields and poorer pregnancy chances in association with IVF.

FSH, even longer than AMH, has been utilized to assess ovarian reserve and predict pregnancy chances [29]. FSH, however, reflects distinctively different stages of follicle maturation [30]. While AMH that is only expressed during the early stages of follicular development, recent animal data suggest that FSH affects practically all stages of follicle maturation [30], though its significance becomes most pronounced during the last stages, the so-called gonadotropin-sensitive phase of folliculogenesis [31]. At such late stages it is quality, and no longer quantity of the follicle pool that is being determined, and oocyte competence becomes the primary issue. The expectation, therefore, would be that, in contrast to AMH, FSH is, overall, more reflective of quality than quantity.

Here reported FSH findings in high responders, and previously reported data in women with diminished ovarian reserve [19], support this hypothesis: While AMHo lost predictive capacity when adjusted for female age, FSHo maintains predictive value for pregnancy. These data suggest that, especially in older women, FSHo may be the better diagnostic parameter in predicting pregnancy chances and confirm previous findings on age-specific discrepancies in total AMH and FSH, where FSH achieved better predictive value above age 42 years [32].

Comparable to AMHo, FSHo, of course, also can be viewed as defining a low and high ovarian phenotype, as, for example, the same total elevated FSH level will result in lower FSHo with higher oocyte numbers, and higher FSHo with fewer oocytes. Since FSHo maintains a negative association with pregnancy chances, the lower FSHo ovarian phenotype would denote better, and the higher FSHo poorer pregnancy odds, as our results show. One, therefore, can hypothesize the existence of four distinct ovarian phenoptypes, based on AMHo and FSHo: (i) high AMHo/high FSHo; (ii) high AMHo/low FSHo; (iii) low AMHo/highFSHo and (iv) low AMHo/lowFSHo. Studies are currently underway to determine whether these ovarian phenotypes correspond with specific patient characteristics seen amongst women with PCOS of different etiologies.

Even though FSHo remained significant after adjusting for potential confounding factors, the rate of useable embryos also demonstrated a significant association with clinical pregnancy, which was independent of FSHo. This may contribute to widely contradictory pregnancy chances reported in high responders. While some authors describe excellent pregnancy potential even after overt clinical ovarian hyperstimulation [33], others report poor pregnancy rates [34], [35].

AMHo and FSHo may, therefore, be used to give high responders a better assumption of their pregnancy chances for their current cycle. Follow up studies are required to assess a likely effect of AMHo and FSHo on pregnancy in cryopreservation cycles and for future IVF attempts. In addition to FSHo’s and AMHo’s clinical impact for patient counseling, it suggests a significant relation of granulosa cell function and pregnancy potential in high responder patients. This may be particularly important for different sub-types of PCOS.

### Limitations and Future Research

The here presented data did not aim to identify different PCOS subtypes. However, our results strongly support the assumption of such phenotypes. Further studies should, therefore, address this question.

In conclusion, here reported results demonstrate in high responder patients a limited, likely age-restricted, predictive significance of AMHo, and age-unlimited predictive value for pregnancy in association with IVF for FSHo, while total AMH and FSH levels fail to show such associations. FSHo, therefore, is confirmed as an, overall, superior prognostic pregnancy parameter over AMHo, as previously also reported in women with diminished ovarian reserve. AMHo and FSHo might, therefore, further enhance our understanding of the components of FOR AMH and FSH best represent.
